# Delivering Bad News: An Approach According to Jewish Scriptures

**DOI:** 10.5041/RMMJ.10154

**Published:** 2014-07-25

**Authors:** Sody A. Naimer, Moshe Prero

**Affiliations:** 1 Eilon Moreh Family Health Center, Shomron, Israel;; 2 Department of Family Medicine, Siaal Family Medicine and Primary Care Research Center, Faculty of Health Sciences, Ben-Gurion University of the Negev, Beer-Sheva, Israel;; 3 Medical School for International Health, Ben-Gurion University of the Negev, Beer-Sheva, Israel

**Keywords:** Bad news, Bible, breaking news, Judaism, oral Jewish scriptures

## Abstract

Despite a preoccupation in the medical literature with developing an effective approach for breaking bad news, the sources are based on personal opinion alone and only in some instances on qualitative research. Recognizing the gravity of this topic coupled with respect for the wisdom of the written and oral Jewish scriptures, this work is an attempt to delve into the diverse ancient writings to draw conclusions regarding a recommended methodology to guide and inform this task.

It is interesting to learn that most elements related to this topic have previously been raised in various forms in the scriptures. The issues range from where, when, and how the bearer of bad news should undertake this duty, to details such as the environment, the format, the speed, and depth of the details to be disclosed. The essence of this paper is to enrich the reader using both positive and negative examples found in the Jewish heritage. Adopting these principles will hopefully provide an effective method for performing this unpleasant obligation, with the goal of limiting harmful consequences as much as possible.

## INTRODUCTION

### Sody A. Naimer: A Personal View

For many years, I have carried the burden of a traumatic experience I had as a young physician. I had been asked to approach a young family to inform a woman whom I had never met that her husband had died in a car accident. I had never received any formal preparation, nor practical or emotional guidance about how to deliver such news. The difficult reaction I witnessed from that young widow caused me to try, as much as possible, to avoid attending situations where bad news had to be delivered. Since then, I have accepted the task of delivering negative medical news as compelling orders to be obeyed, albeit unwillingly. I felt that society had placed this unpleasant task upon the physician simply because there was no other appropriate party to call on (rarely do medical consequences result from these encounters).

My personal approach to this subject dramatically changed when Professor Joseph Herman (my mentor) shared a story with me. Once, someone called him mistaking his number for that of a different doctor with the same name. The caller told Professor Herman that he was trying to locate the physician of a patient who had just passed away. The patient’s family had to be notified of the death, and the physician was nowhere to be found. After hearing this, Professor Herman kindly volunteered to notify the family, despite the fact that he had never been involved in the case.

This story changed my approach to delivering bad news. I started to view the task as a genuinely good deed, an opportunity to limit the intensity of the blow. It could be informing someone that their loved one had just passed away under tragic circumstances, breaking the news of a diagnosis of illness to a patient, or giving other shocking news to an unsuspecting family member. The manner in which the news is delivered can make the difference between solidarity and partnership, on the one hand, and alienation and perhaps even hostility, on the other. It is a difference between enhancing confusion, uncertainty, or absolute helplessness and, instead instilling a feeling of acceptance that may lead to meaningful action. It is the difference between creating an atmosphere of emptiness and despair, and setting a challenge with a sense of sacrifice.

### Searching for Guidance

In the medical literature, much attention has been given to this issue since its impact is pervasively recognized.^1^–^3^ There is little evidence about the best methods for giving bad news, so most guidelines are based on opinion.^4^,^5^ Elisabeth Kubler-Ross provided an unsurpassed contribution on this subject as the result of her countless hours of interviews with patients who had been informed that they had terminal cancer. She formulated a theory that included the phases of emotional stages that a person goes through the first time they receive the news that their illness is terminal.^6^ This theory has stood the test of time, and experience has shown that most people in similar situations react according to the stages she described years ago. These stages include: denial, anger, bargaining, depression, adaptation, and acceptance. These responses are highly personal and can occur in any order and combination and over any time interval. It is apparent that intensive support to escort the subject through and beyond these stages will make it easier and quicker to arrive at the ultimate stage of acceptance of the new reality. Therefore, it is imperative at the outset of the process to take actions to prevent fixation at an early stage, such as denial or anger, as will be illustrated below.

As practicing orthodox Jewish physicians, efforts to consolidate our own personal styles led to the exploration of all possible sources of our tradition in order to make it easier to perform this duty to the best of our ability.

This paper quotes and analyzes several events recorded in the Bible and Talmud (Jewish oral scripture) in order to draw lessons from our forefathers and to witness for ourselves a divine approach for how such situations should be handled. The goal is not to dictate specific behavior, but rather to judge the source and to consider whether we may conduct ourselves similarly under such circumstances. In general, the objective is to formulate a response comprised of a combination of hope, understanding, sensitivity, empathy, and sympathy.

## EXAMPLES FOR BREAKING BAD NEWS IN JEWISH SCRIPTURES

The chosen sources are organized in chronological order as they appear in the scriptures and not necessarily conceptually according to importance or as a theme. Both positive and negative examples are given so that we can see different aspects of how a difficult message should or should not be conveyed.

### Positive Examples of Breaking Bad News

#### 1. Genesis 3:9–10

And the LORD God called unto the man, and said unto him: “Where art thou?” And he said: “I heard Thy voice in the garden, and I was afraid, because I was naked; and I hid myself.”

Firstly, it is important to create an environment that allows for communication to take place before delivering the content of the message. This explains God’s question to Adam. It was necessary to engage him in conversation. Contact was generated by asking a general question; only after a verbal exchange regarding Adam’s hiding did God deliver the news that Adam had been caught sinning. As a consequence it was decreed that Adam would eventually die.

#### 2. Genesis 27:31–33

And he also made savoury food, and brought it unto his father; and he said unto his father: “Let my father arise, and eat of his son’s venison, that thy soul may bless me.” And Isaac his father said unto him: “Who art thou?” And he said: “I am thy son, thy first-born, Esau.” And Isaac trembled very exceedingly, and said: “Who then is he that hath taken venison, and brought it me, and I have eaten of all before thou camest, and have blessed him? yea, and he shall be blessed.”

Instead of directly notifying Esau of the actions of his brother, the father leads his son to the difficult conclusion that his brother deceived him. He softens this difficult reality by bestowing a smaller blessing upon him, and concludes by apologizing that there is nothing more that he can do.

#### 3. Genesis 31:4–7

And Jacob sent and called Rachel and Leah to the field unto his flock, and said unto them: “I see your father’s countenance, that it is not toward me as beforetime; but the God of my father hath been with me. And ye know that with all my power I have served your father. And your father hath mocked me, and changed my wages ten times; ...”

There are a number of observations that can be drawn from this story. The context here is that Jacob was seeking a secluded location where he could reveal to his family his intent to rise up against his father-in-law Laban, thereby endangering himself, them, and all his possessions. The location of this conversation is noteworthy. It transpires in a private, quiet, faraway place, where the lengthy detailed speech is delivered out of ear-shot, safely, and in confidence. This allows the message to be presented in its entirety, starting with past events and continuing to the present circumstances. This way Jacob conveys his opinion in such a way that his wives are able to arrive at the same conclusion—there is no other reasonable option at this point than to escape.

#### 4. Genesis 37:32

and they sent the coat of many colours, and they brought it to their father; and said: “This have we found. Know now whether it is thy son’s coat or not.”

Despite the negativity of the sons’ deeds, as far as “getting the information across” there is a constructive lesson to learn from this story. Here we see that, aside from the desire of Joseph’s brothers to avoid a direct lie, they begin by using visual aids, the clothes of Joseph, that unquestionably represent Joseph exclusively. Secondly, they refrain from making any explicit statements. They lead Jacob to the realization that plunges him into a cycle of mourning: “Joseph was devoured.”

#### 5. Genesis 38:25–26

When she was brought forth, she sent to her father-in-law, saying: “By the man, whose these are, am I with child”; and she said: “Discern, I pray thee, whose are these, the signet, and the cords, and the staff.” And Judah acknowledged them, and said: “She is more righteous than I;”

Here we see another example where, instead of verbal communication, there is the presentation of an object that is capable of telling the whole story, leading the subject to his eventual realization, at his own pace. In this case, the seemingly wayward daughter-in-law was dismissed from being punished for her attempt to re-attach herself to her deceased husband’s family.

#### 6. Genesis 45:25–27

And they went up out of Egypt, and came into the land of Canaan unto Jacob their father. And they told him, saying: “Joseph is yet alive, and he is ruler over all the land of Egypt.” And his heart fainted, for he believed them not. And they told him all the words of Joseph, which he had said unto them; and when he saw the wagons which Joseph had sent to carry him, the spirit of Jacob their father revived.

The Midrash (Sechel Tov, Genesis 45) comments that the brothers did not tell Jacob directly. They had the message delivered via Asher’s daughter, Serach. The Rabbis in the Talmud relate the tradition that Serach gradually conveyed to Jacob the news that Joseph was still alive by singing songs to herself to that effect, in his presence, for a period of weeks and months. The purpose was that when the time came to deliver to Jacob the long-hoped-for and unexpected message that Joseph was alive, he would then be capable of hearing this without experiencing any unnecessary shock. Ultimately, this would allow Jacob to accept the news, and restore him to his pre-mourning vitality.

#### 7. Exodus 32:17–19

And when Joshua heard the noise of the people as they shouted, he said unto Moses: “There is a noise of war in the camp.” And he said: “It is not the voice of them that shout for mastery, neither is it the voice of them that cry for being overcome, but the noise of them that sing do I hear.” And it came to pass, as soon as he came nigh unto the camp, that he saw the calf and the dancing; and Moses’ anger waxed hot, and he cast the tablets out of his hands, and broke them beneath the mount.

Joshua relates to Moses that a uniquely suspicious celebration was coming from the Israelite encampment at the foot of the mountain. Moses did not react immediately. Instead, he waited to see the golden calf with his own eyes before breaking the tablets. Perhaps Moses’ hesitation to break the tablets came from the fact that he was reluctant to rely on the initial report because it came from someone in a position of lesser authority. Furthermore, circumstantial evidence (the sight of joyous idolatry), internalized by other senses, may register more convincingly than listening to the account of events.

#### 8. Deuteronomy 9:11–17

And it came to pass at the end of forty days and forty nights ... And the LORD said unto me: “Arise, get thee down quickly from hence; for thy people that thou hast brought forth out of Egypt have dealt corruptly; they are quickly turned aside out of the way which I commanded them; they have made them a molten image.” ... So I turned and came down from the mount, and the mount burned with fire; and the two tables of the covenant were in my two hands ... And I looked, and, behold, ye had sinned ... And I took hold of the two tables, and cast them out of my two hands, and broke them before your eyes.

The Lord Himself informs Moses that the nation has sinned, and yet, Moses does not react until he himself sees it. We can infer from this that at times, in the absence of supporting evidence, the receiver of bad news, while not casting doubt on the veracity of the message, will simply refuse to believe it until he sees it with his own eyes.

#### 9. Numbers 27:12–14

Get thee up into this mountain of Abarim, and behold the land which I have given unto the children of Israel. And when thou hast seen it, thou also shalt be gathered unto thy people, as Aaron thy brother was gathered; because ye rebelled against My commandment in the wilderness.

Firstly, Moses is commanded to perform an action: to ascend the mountain and view the Holy Land. Slowly Moses is given the opportunity to understand and internalize his punishment. The Lord consoles Moses by giving him a welcomed piece of news that he will die in the same preferred manner that his brother Aaron died. This way Moses received the reassurance that he had hoped for. It is only at the end, when the full consequence of this message is conveyed, that Moses is rebuked for his sin.

#### 10. Judges 19:29

And when he was come into his house, he took a knife, and laid hold on his concubine, and divided her, limb by limb, into twelve pieces, and sent her throughout all the borders of Israel.

Here we witness a disturbing example of a desperate act intended to shock any sane person and to demonstrate that a terrible crime has been committed against a human being. This news was delivered via action rather than words, in a manner that clearly cries out that all borders of acceptable behavior have been breached and an abominable act has been committed in Israel.

#### 11. I Samuel 3:11 and 16–18

And the LORD said to Samuel: “Behold, I will do a thing in Israel, at which both the ears of every one that heareth it shall tingle” ... Then Eli called Samuel, and said: “Samuel, my son ... I pray thee, hide it not from me ...”

Despite Samuel’s lack of desire to be the one to deliver bad news, the individual appointed to fulfill this task should not evade this responsibility out of fear of repercussions. Instead, the message should be conveyed in its entirety, as eventually transpired in this case.

#### 12. I Samuel 4:12, 17–18

And there ran a man of Benjamin out of the army, and came to Shiloh the same day with his clothes rent, and with earth upon his head ... “Israel is fled before the Philistines, and there hath been also a great slaughter among the people, and thy two sons also, Hophni and Phinehas, are dead, and the ark of God is taken.” And it came to pass, when he made mention of the ark of God, that he fell from off his seat backward by the side of the gate, and his neck broke, and he died …

Eli the High Priest (Kohen) was an elderly, blind, and fragile 98-year-old. The first thing he hears is the noise of screaming. We find that the messenger’s opening words elaborate upon extraneous details that have little to do with the important main message. However, this plays the important role of opening communication with Eli, and allows Eli to ask for more details. Following this exchange, the messenger delivers his message starting with the least difficult news. The first message delivered is generally about the battle, then progresses to the fate of Eli’s sons, and finally the most severe news, judging by Eli’s reaction, of the capture of the ark. Remarkably the fall of his sons is told before the “taking” of the ark to which Eli succumbs. This is a reversed order compared to the sequence of events the narrator accounts earlier and reveals the wisdom of the informer.

#### 13. I Samuel 25:36, 37

And Abigail came to Nabal; and, behold, he held a feast in his house, like the feast of a king; and Nabal’s heart was merry within him, for he was very drunken; wherefore she told him nothing, less or more, until the morning light. And it came to pass in the morning, when the wine was gone out of Nabal, that his wife told him these things ...

Abigail finds that there is no room for delivering bad news when the intended recipient is not of a clear mind, neither ready nor able to absorb disturbing information. Perhaps the information will be partially understood or perhaps not at all. Therefore she waits until the recipient has exited his nebulous state and returns to his full faculties. Only then will he be capable of listening and internalizing the implications of what she will tell him.

#### 14. II Samuel 12:15, 18–20

And the LORD struck the child that Uriah’s wife bore unto David, and it was very sick ... And it came to pass on the seventh day, that the child died. And the servants of David feared to tell him that the child was dead; for they said: “Behold, while the child was yet alive, we spoke unto him, and he hearkened not unto our voice; how then shall we tell him that the child is dead, so that he do himself some harm?” But when David saw that his servants whispered together, David perceived that the child was dead; and David said unto his servants: “Is the child dead?” And they said: “He is dead.” Then David arose from the earth, and washed, and anointed himself, and changed his apparel …

We observe again that David’s servants try to avoid telling him that which he is afraid of hearing. Their apprehension is verbalized, perhaps “he will do something terrible.” There is no description of what that terrible thing may be, nor to whom it may have been directed. However, the message arrives indirectly; David understands when he hears the others whispering. It is then that he asks a direct question regarding the well-being of his child, and they confirm his suspicions.

#### 15. II Kings 4:27

And when she came to the man of God to the hill, she caught hold of his feet. And Gehazi came near to thrust her away; but the man of God said: “Let her alone; for her soul is bitter within her; and the LORD hath hid it from me, and hath not told Me.”

In the dreadful situation in which the Shunamite woman finds herself, suffering the unbearable loss of her only child, she crosses the norms of modest behavior. Despite this, the prophet is sensitive to her extremely difficult situation, and allows her to behave temporarily in an exceptional manner and fall to his feet. The prophet slightly calms his petitioner by showing that he understands her pain, and that her pain takes precedence over all else. His reaction is an urgent response, and by such action he instills a sense of calmness and trust.

#### 16. Job 1:14–18

“The oxen were plowing, and the asses feeding beside them; and the Sabeans made a raid, and took them away; yea, they have slain the servants with the edge of the sword; and I only am escaped alone to tell thee.” ... While he was yet speaking, there came also another, and said: “Thy sons and thy daughters were eating and drinking wine in their eldest brother’s house; And, behold, there came a great wind from across the wilderness, and smote the four corners of the house, and it fell upon the young people, and they are dead; and I only am escaped alone to tell thee.”

It is notable in this description of the delivery of bad news that despite the purpose of these events to test Job, the messages are delivered in a specific order. They start with the story of the animals grazing in the field being destroyed by an armed enemy. The news is delivered in a manner progressing from least to most personal, starting with property, then servants, and then children. This illustrates the principle of breaking bad news gradually. Additionally, this case provides another example of successive messengers, removing all doubt and uncertainty regarding the authenticity of the information conveyed.

#### 17. Babylonian Talmud, Tractate Yevamot (page 16)

In the days of Rabbi Dossa son of Horkinus the daughter’s husband’s second wife was permitted to the brothers. This was a difficult thing for the wise men because he was a great Rabbi whose eyes could not bring him to appear at the learning hall. They said who will go and notify him? Rabbi Joshua said: “I will go!” After him Rabbi Eliezer son of Azarya. And after him Rabbi Akiva went, and they stood at the entrance to his house. His servant entered and told him: “Rabbi, the wise of Israel have come to you.” He said to her: “Let them enter” and they entered and took hold of Rabbi Joshua and sat him down on a bed of gold. He said: “Tell your other pupil to sit.” He said: “Who is he?” “Rabbi Eliezer son of Azarya” and they sat him down on a bed of gold. He said: “Tell your other pupil to sit.” He said: “Who is he?” “Rabbi Akiva son of Joseph whose name is famous from one end of the world to the other.” “Sit my son, sit, there should be a multitude of your kind in Israel ...” They began to encircle him with different laws until they reached the “daughter’s husband’s second wife.” They said to him: “What is the ruling on this?” He told them: “It’s disputed between the houses of Hillel and Shamai.” “And the verdict is with who?” He said: “As house of Hillel.” They said: “And behold in your name they told in that the ruling is with the house of Shamai ...”

This source teaches us the importance of choosing the most qualified person to inform Rabbi Dosa that he is eccentric in his ruling. Therefore they approached a number of the generation’s leaders to volunteer. The technique chosen is through a candid conversation until the intended topic is reached. At the end, Rabbi Dosa explains that in fact, it was not he who issued the disputable ruling discussed. This was a case of mistaken identity, as the real source of that legal decision was Rabbi Dosa’s younger brother Jonathan son of Horkinus. We see the importance of verifying the identity of the individual to whom the bad news must be delivered.

#### 18. Babylonian Talmud, Tractate Bava Metzia (page 59)

They say, on that very day, all the purities purified by Rabbi Eliezer were burned in fire and they unanimously ex-communicated him and cursed him. They asked: “Who will go and notify him?” Rabbi Akiva told them: “I will go, lest an unworthy person will notify him and this may destroy the whole world”. What did Rabbi Akiva do? He attired in black and wrapped himself in black and sat before him four cubits away. Rabbi Eliezer said to him: “Akiva what is one day from the next?” “Rabbi” he answered, “I think your contemporaries are keeping their distance from you ...” Even he rent his clothes and removed his shoes and descended to sit on the ground, his eyes filled with tears ...

Here we learn of the tremendous impact the delivery of the news itself can have. Harm can be caused with very diverse repercussions. We find that the most senior and qualified sage volunteered to carry out this service. In Rabbi Akiva’s particular manner of dress and speech he leads Rabbi Eliezer to initiate verbal communication and ultimately to perceive that he has been ex-communicated.

### Negative Examples for Breaking Bad News

#### 19. II Samuel 1:2, 10–12

… a man came out of the camp from Saul with his clothes rent, and earth upon his head; and so it was, when he came to David, that he fell to the earth, and prostrated himself … “So I stood beside him, and slew him, because I was sure that he could not live after that he was fallen; and I took the crown that was upon his head, and the bracelet that was on his arm, and have brought them hither unto my lord.” Then David took hold on his clothes, and rent them; and likewise all the men that were with him. And they wailed, and wept ...

The messenger in this case did not make the effort to clarify the nature of the sensitive relationship between the receiver of the news and his perceived enemy. Immediately following a short dialogue, the messenger delivers the news of the death of Saul and his son in a crude manner, without any details. David reacts by tearing his clothes in a sign of mourning only after all of the details have emerged, and only then does he see with his own eyes the tangible proof that supports the message of the Amalekite messenger. This testifies to the phenomenon of denial, that the receiver of bad news is unable to come to terms with the news of the death of a loved one, or family member. The bitter end of this case concludes with the execution of the messenger due to his active participation in the killing of Saul.

#### 20. II Samuel 18:19–20, 32–19:1

Then said Ahimaaz the son of Zadok: “Let me now run, and bear the king tidings, how that the LORD hath avenged him of his enemies.” And Joab said unto him: “Thou shalt not be the bearer of tidings this day, but thou shalt bear tidings another day; but this day thou shalt bear no tidings, forasmuch as the king’s son is dead.” ... “Is it well with the young man Absalom?” And the Cushite answered: “The enemies of my lord the king and all that rise up against thee to do thee hurt, be as that young man is.” And the king was much moved, and went up to the chamber over the gate, and wept ...

Twice Joab tries to dissuade the volunteer from performing the task of informing the father of the death of his son, but Ahimaaz persists. We find in this story the commonly encountered avid enthusiasm of individuals to rush the knowledge of a dire event to involved parties. Here the messengers make critical mistakes when they relay the news from the battle field. They do not appreciate the deep emotional attachment that David still had for his disobedient son Absalom. Ahimaaz softens his delivery when asked about Absalom, reporting that there was commotion but evades the question as to the fate of Absalom. The Cushite then delivered his tidings devoid of emotional sensitivity, and in fact, with overtones of joy. He praises both the outcome of the battle and the killing of Absalom. David’s comprehension of the death of his son is based on the fact that he received two reports, one after another, that supported each other. Here, too, David’s tears, followed by anger, are delayed precisely until he hears the end of the second account.

## GENERAL RECOMMENDATIONS

Based on these examples it is possible to provide general recommendations regarding the delivery of bad news (the numbers in brackets refer to the examples above):

- It is preferable for the informer to be emotionally close to the recipient, and that he or she understand the full details of the message to be delivered. The informer should also ascertain that he or she is the most qualified individual to deliver the message, before undertaking the mission [3,11,16,18–20].
- If present, one should overcome enthusiasm to deliver the bad news. Rather, the facts should be verified, and only then, with certainty that the news must be delivered, should the task be undertaken. Even then, the optimal timing of the message should be considered, and the message should only be delivered when the recipient is best able to hear it [3,11,13,16,19,20].
- Prevailing stresses and strains should not be allowed to coerce immediate delivery of the news or to interfere in the process of accurate message delivery, unless there is no alternative [3,14,16,18–20].
- A time should be chosen when the recipient is free from distraction and prepared to hear the news. The news should not be delivered when the recipient is not in a clear state of mind, or is busy with other necessary activities [3,13,14].
- The message should be delivered in a confidential, private setting, conducive to open communication. The recommended location is one that permits an emotional response, without undue exposure of the recipient [3,18].
- Communication should first be established in ordinary conversation. The recipient should be engaged and allowed to respond to questions verbally, before actually hearing the bad news [1,9,12,15,18–20].
- The opening of the conversation should be indirect or general and should gradually lead to the bad news. Therefore, one should begin with details that are easier to digest and then lead to the more difficult news [1– 5,9,12,18,19].
- When possible, it is preferable to allow the recipient to arrive at the conclusion on their own, even if this results in a prolonged process to allow the recipient to ask questions [3–6,9,10,14,17,18].
- The tone, language, and style of delivery should be adapted to the subjective norms of the recipient [3,6,17].
- One should be sensitive to the recipient. Sight, body language, and the behavior of people in the surrounding area are all props that support the message being delivered, and should all be utilized. Multiple messengers should be involved in delivering the message to help the recipient accept the message as truth [4–10,12,14,18,20].
- On the one hand, the bearer of the news should be a full partner in the emotional distress that is caused by the new reality. However, he/she should leave the recipient with some hope, support, and security that they possess the necessary strength to cope with the new situation and continue with life [2,3].
- At the end of the encounter, ensure that the message delivered was clear, not confusing, and not open to different interpretation; verify that the main message was understood [3,6].
- Allow for flexibility in the reaction of the recipient, even the more exceptional ones. Be prepared for hazardous or dangerous reactions, and try to be patient, as much as possible, with unexpected behavior [14,15].

## CONVEYING A MESSAGE IN PRACTICE

Finally, we will examine two more classic examples, each illustrating the extremes of how difficult news should be delivered. The first example is negative, the second is positive.

### Sefer Hayashar (Parshat Veyaira) [Pirkei Rabbi Eliezer 32]

And Satan went to Sarah appearing as a decrepit, old man, very humble while Abraham is still sacrificing to the Lord. And he said to her: “You must know the whole story of what Abraham did to Isaac today, that he took Isaac and built an altar and slaughtered him and sacrificed him on the altar, and that Isaac was screaming and crying before his father and there was no one to watch over him or sympathize with him.” And Satan elaborated upon this to Sarah repeatedly and he left and Sarah heard all of Satan’s words and she thought that he was an old man of people that were by her son that came and told her such things. And Sarah raised her voice and wept and cried loudly and bitterly and she threw herself to the ground, and threw dust on her head, and said: “My son Isaac, if only I were to die myself instead of you this very day.” She continued to weep and said: “I am filled with sorrow that I raised you and cared for you, all my joy turned to mourning.” After that she rose, she went this way and that way through barren land, and went to Hebron and asked all the passersby that she met on her way and they could not respond to her words to say what happened with her son. And she arrived with her servants and maids at Kiryat Arba that is Hebron, and asked about her son and she sat there, and sent her servants to request where did Abraham and Isaac go? And they went to seek them by Shem and Eiver and could not find them, and they searched all over the land to no avail. Behold, the Satan revealed himself to Sarah as a man and he came and stood in front of her and said to her: “I lied to you, Abraham did not slaughter Isaac his son and he did not die.” And it was that as she heard this, she became very very joyful about her son and her soul departed from joy and she died and was collected to her nation.

Satan is self-revealed as a total stranger, with no specific identity or connection to Sarah. There was no opportunity to verify the facts or the reliability of their source. There is no communication between the partners at this opportune meeting. The message is given without details or background information. The deliverer of the message suddenly disappears after delivering the false message. Worst of all, the recipient is left alone without refuge; helpless, wandering here and there to confirm her suspicions. While still dealing with her doubts, the same doubtful source delivers the exact opposite message, causing her death. One’s naivety rebels against the intensity of such cruelty, being so abused and susceptible to bad news in such a manner.

On the other hand, our last example displays the principles which we summarized in a clear and well planned, most empathetic manner. Agreeably, this is the best way to deliver news of a heavy loss. This illustrates that with understanding and the necessary sensitivity, it is possible to perform the task well and mildly, gradually exposing the full scope of the tragedy.

#### Midrash Raba, Proverbs (31:10)

“A woman of valour who can find” (Proverbs 31:10) It is told about Rabbi Meir that as he sat learning in the study hall at “mincha time” on Sabbath his two sons had died and his wife placed them both on the bed and spread a sheet upon them. After Sabbath Rabbi Meir returned home from learning and asked her where the boys were. She told him that they went to learn. He said he looked for them there but he could not find them. He was given a cup of wine for the havdala and he made the blessing. Again he asked about their whereabouts, she told him they went to another place and that they are coming soon. She served her husband a meal and he ate and blessed and after the blessing she asked him: “May I ask you one question?” He told her: “Please ask your question” and she said: “Rabbi, yesterday a man gave me a deposit and now he has come to claim it back, must we return it or not?” “My daughter” he said “He who has the deposit must return it to its owner.” She said “Rabbi, without your consent I would not have delivered unto him.” What did she do? She took him by the hand and escorted him to that room and brought him near the bed and she raised the sheet from them. And he saw them both dead lying on the bed. He started to weep and cried: “My sons, my sons, my Rabbis, my Rabbis; sons as the course of nature, and my Rabbis—as you enlightened me by your Torah!” At that instance she said to Rabbi Meir: “Rabbi, was not that what you said to me, that I must return the deposit to its owner?” He said: “The Lord giveth and the Lord taketh away” (Job 1:21). Rabbi Chanina said: This way she consoled him and managed to reassure him, therefore it says: “A woman of valour who can find?”

## CONCLUSION

As the bearer of bad news, the physician is drawn into a human drama that contradicts “*Primum non nocere*,” one of the principal bioethic precepts that we learn as medical students. Delivering bad news often shocks the recipient(s), burdening the messenger with the difficult task to help them recover and cope with the emotional pain of a new and unwished-for reality.

In this paper, drawing from Jewish sources, we present the elements: attention to the environment, the message format, the pace of the explanation, and the extent of details to be disclosed as principles with which to shape a methodology. It is hoped that this will help reduce the dread of delivering bad news for the benefit of those who receive it.
